# Supplementary material for: MetaRibo-Seq measures translation in microbiomes
Source: Nat Commun. 2020 Jun 29;11:3268. doi: 10.1038/s41467-020-17081-z (PMC7324362; doi:10.1038/s41467-020-17081-z)
Supplement: Supplementary file 10 — Supplementary Data 7 [file 41467_2020_17081_MOESM10_ESM.zip › File2/Confidence_High_Taxonomy/84458_out.krona.html]

Javascript must be enabled to view this page.

members
magnitude
magnitudeUnassigned
count
unassigned
taxon
rank

84458\_out

7

superkingdom
2
7

1239
7
phylum

class
186801
7

186802
7
order

2
31979
family

genus
2
1485


SRS144135\_contig\_number\_28847
1
1262815
species

species

SRS024435\_contig\_number\_30485
1262841
1

1898207
5

SRS013476\_contig\_number\_40363SRS024132\_contig\_number\_1984SRS053356\_contig\_number\_contig-100\_29835.115871SRS146812\_contig\_number\_48044SRS150029\_contig\_number\_2634
species
